# Oral GLP-1 analogue: perspectives and impact on atherosclerosis in type 2 diabetic patients

**DOI:** 10.1186/s12933-021-01417-0

**Published:** 2021-12-15

**Authors:** José Francisco Kerr Saraiva, Denise Franco

**Affiliations:** 1 Faculdade de Medicina do Centro de Ciências da Vida - Pontifícia, Universidade Católica de Campinas, Av John Boyd Dunlop, s/n - Jd. Ipaussurama, Campinas, SP CEP: 13060-904 Brazil; 2 CPCLIN/DASA Centro de Pesquisas Clínicas, Av Angelica, 2162 - Consolação, São Paulo, SP CEP: 01228-200 Brazil

**Keywords:** Glucagon-like peptide 1, Oral semaglutide, Diabetes, Atherosclerosis, Cardiovascular disease, Stroke prevention

## Abstract

Cardiovascular events related to atherosclerosis are responsible for high morbidity and mortality among patients with type 2 diabetes. Improvement in care, especially in early stages, is crucial. Oral semaglutide, a glucagon-like peptide 1 analogue, controls blood glucose and results in significant body weight loss in patients with type 2 diabetes. Beyond these well-known effects, an interesting aspect of this drug is its antiatherogenic activity, which should be further explored in clinical practice. This paper reviews the evidence related to oral semaglutide decreasing cardiovascular risk in patients with type 2 diabetes, focusing on the drug’s antiatherosclerotic properties. The glucagon-like peptide 1 analogue restores endothelial dysfunction, induces vasodilatation, and reduces plasma lipids. Oral semaglutide showed cardiovascular safety profile, with significant reduced risk of death from cardiovascular events. Based on current data, clinicians should consider oral semaglutide for type 2 diabetes management.

**Supplementary Information:**

The online version contains supplementary material available at 10.1186/s12933-021-01417-0.

## Introduction

Type 2 diabetes mellitus (T2DM) is a prevalent disease with the potential to become a pandemic and one of the leading causes of mortality worldwide [1, 2]. More than 400 million people worldwide have T2DM [3], and its incidence increases as the population ages, obesity increases, and urbanization progresses [4–7].

The most prevalent cardiovascular complication of T2DM is related to atherosclerosis and its complications, such as acute myocardial infarction, stroke, and peripheral artery disease, which are responsible for high mortality and morbidity among T2DM patients. In a recent large cross-sectional study evaluating 9823 T2DM patients from around the world (including Brazil), 34.8% of patients had established cardiovascular disease (CVD), of whom 85.8% had atherosclerotic cardiovascular disease (ASCVD) [8]. Among the 43.9% of the study population composed of Brazilian patients (n = 912), 85.8% had ASCVD [9]. The prevalence of coronary heart disease (CHD) was 27.9%; 8.7% presented cerebrovascular disease, and 3.4% presented carotid artery disease [9]. ASCVD quadruples the risk of CHD, doubles the risk of stroke, and triples the risk of death [10]. T2DM generates an environment prone to atherogenesis. Chronic hyperglycaemia, the production of reactive oxygen species (ROS), and the release of inflammatory cytokines are some of the factors causing endothelial dysfunction in patients with diabetes [11, 12]. This endothelial dysfunction decreases the potent vasodilator nitric oxide (NO) in endothelial and vascular muscle smooth cells, increases the levels of vasoconstrictors such as endothelin-1 [13], and creates a hypercoagulable state by activating platelet aggregation and inhibiting fibrinolysis [14]. Compared to individuals without diabetes, patients with diabetes present a higher atheroma volume, a smaller arterial lumen, more rapid progression of atherosclerosis, more macrophage accumulation, and a higher incidence of thrombus [15, 16]. A study examining coronary atherectomy specimens showed that 62% of patients with T2DM presented coronary thrombus compared with 40% of patients without diabetes (p = 0.04) [16].

Since the discovery of insulin, the main goal of diabetes treatment has been the control of glycaemia to prevent complications. Despite the benefits observed in adequate glycemic control in reducing microvascular events the results are controversial in regard to atherosclerotic disease [17]. Therefore, advances in T2DM therapies are crucial, especially in the early stages of the disease; treatment must effectively decrease the risk before atherosclerosis is established.

Questions regarding the cardiovascular safety of drugs prescribed for T2DM arose as individuals treated with sulfonylureas and insulin showed an increased incidence of major adverse cardiovascular events (MACEs: death from cardiovascular causes, nonfatal myocardial infarction, or nonfatal stroke) [18, 19]. The withdrawal of approval of rosiglitazone due to the increased risk of cardiovascular events [20] resulted in a demand for studies demonstrating cardiovascular safety for any new antidiabetic drug. Consequently, a new era of studies assessing cardiovascular outcomes began showing that some drugs are not only safe but also decrease the incidence of CVD [21, 22].

Glucagon-like peptide 1 (GLP-1) is an incretin 30 amino-acid peptide hormone produced in hindbrain neurons and in specialized enteroendocrine cells (L cells) in the distal small and large intestines that is released after food intake [23, 24]. Glucagon-like peptide 1 receptor (GLP-1R) is present not only in the central nervous system and gastrointestinal tract but also in the pancreas, kidney, lungs, cardiomyocytes, vascular smooth muscle cells, and endothelium [25–29]. GLP-1 acts on pancreatic cells by inhibiting the alpha cells responsible for glucagon secretion [30] and stimulating insulin production in beta cells in response to elevated blood glucose levels [23]. This combined mechanism of action of GLP-1 makes GLP-1 attractive for the treatment of T2DM.

### GLP-1 analogues

GLP-1 analogues were first approved for the treatment of T2DM in 2005. Peptide drugs are highly specific, with less toxicity and fewer drug interactions. However, only the injectable form has been available because peptide drugs are usually not suitable for administration via the oral route, as they present low oral bioavailability, are inactivated when they reach the gastrointestinal tract, and have low rates of diffusion into the cell [31, 32].

Recently, the first orally administered GLP-1 analogue was approved for the treatment of T2DM. The permeation enhancer sodium *N*-[8-(2-hydroxybenzoyl)amino] caprylate (SNAC) co-formulated with semaglutide prevents enzyme degradation, increases its absorption in the stomach, and results in suitable bioavailability [33, 34]. The Peptide Innovation for Early Diabetes Treatment (PIONEER) programme consisted of eight phase 3, randomized, controlled clinical studies and showed that oral semaglutide reduces glycated haemoglobin (HbA1c) and body weight in patients with T2DM and has potential cardiovascular benefits [35–42].

### Semaglutide and clinical atherosclerotic cardiovascular event data

**Fig. 1:**
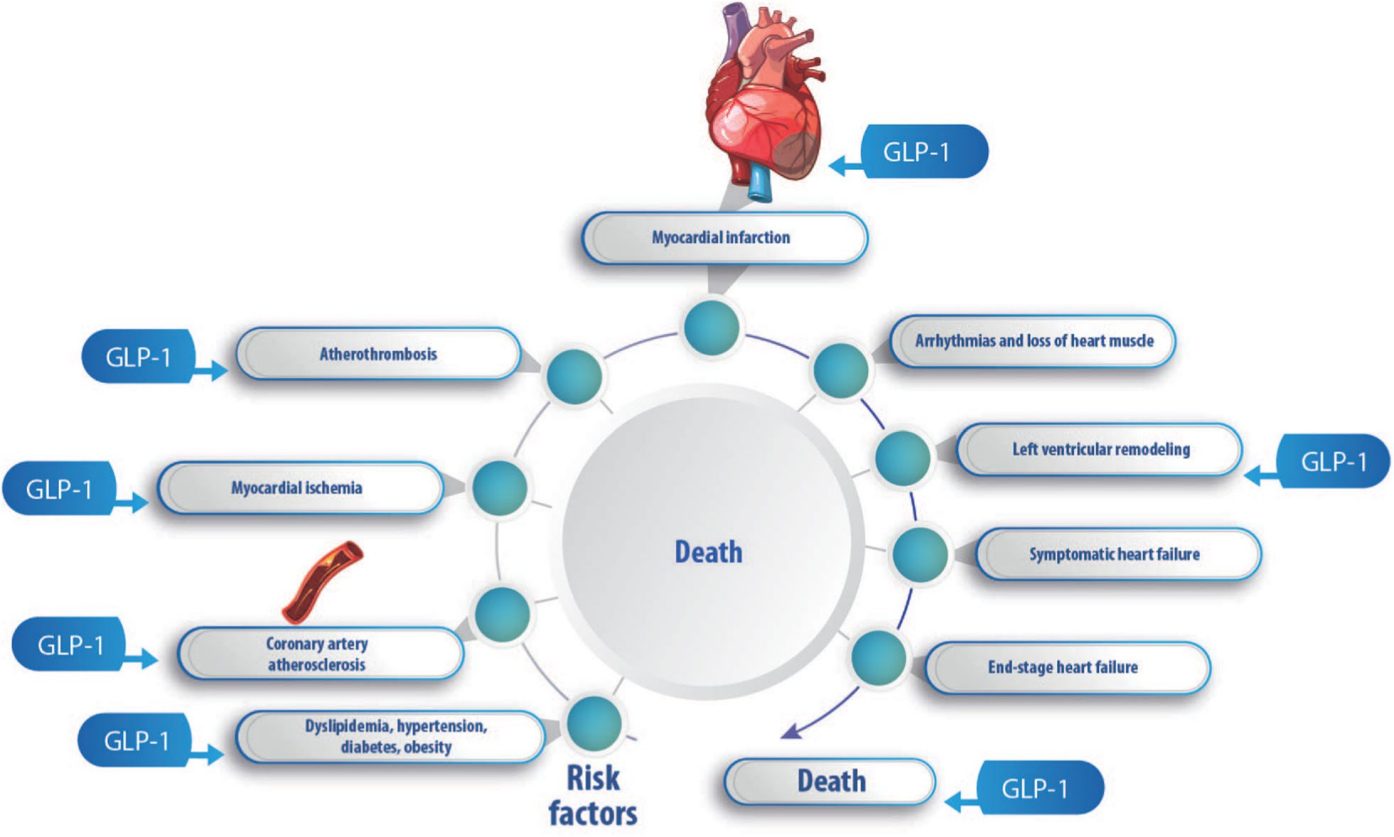
Cardiovascular disease continuum-GLP-1 action. Adapted from [46]

Regulatory authorities recognized that GLP-1 analogues reduce the incidence of MACEs in T2DM patients, but this is only the case for GLP-1R agonists with a molecular structure based on endogenous GLP-1 (semaglutide, liraglutide, albiglutide, and dulaglutide), as they are able to reduce the relative risk of MACEs by up to 10% [43]. International guidelines recommend the use of GLP-1 analogues in T2DM patients with ASCVD or who have high cardiovascular risk [44, 45]. Moreover, considering the CVD continuum where it progresses from risk factors such as diabetes to atherosclerosis and CHD, until it leads to heart failure or death [46], GLP-1 analogues act in different stages of this pathophysiological process (see Fig. 1) without increasing the incidence of arrhythmia or hospitalization or worsening heart failure [22, 40, 47–54].

### Cardiovascular clinical data with oral semaglutide

Data from cardiovascular events including death were collected during the PIONEER program. A summary of all the CV events occurring during these trials are presented in Additional file 1: Table S1. In addition, a cardiovascular outcome trial (CVOT) was also specifically carried out, the PIONEER 6, to evaluate cardiovascular events in a larger population using oral semaglutide [40].

The PIONEER 6 study included 3183 T2DM patients who had ASCVD or who were at high risk, of whom 1591 were treated with oral semaglutide and 1592 were treated with placebo for a median time of 15.9 months [40]. This study was designed to evaluate the cardiovascular safety of oral semaglutide, and the results confirmed the safety, showing the noninferiority of oral semaglutide over placebo in regard to the timing of the first MACE. Nonfatal myocardial infarction occurred in 2.3% of patients treated with oral semaglutide and 1.9% treated with placebo (HR 1.18, 95% CI 0.73–1.90), nonfatal stroke occurred in 0.8% versus 1.0% treated with placebo (HR 0.74, 95% CI 0.35–1.57), and death from cardiovascular causes occurred in 0.9% versus 1.9% treated with placebo (HR 0.51, 95% CI 0.31–0.84). Long-term data are needed to confirm the cardiovascular benefits of oral semaglutide. The Heart Disease Study of Semaglutide in Patients with Type 2 Diabetes (SOUL) is a more extensive and extended phase 3 CVOT presently being conducted, and results are expected in 2024 [55]. It is a double-blind, placebo-controlled study (n = 9642 T2DM patients), assessing the use of oral semaglutide once a day (up to 14 mg) during 3.5–5 years, evaluating the cardiovascular benefit of oral semaglutide [55].

According to a meta-analysis evaluating 453 trials and 21 antidiabetic treatments, oral semaglutide reduces all-cause mortality and cardiovascular death, with the lowest odds ratios (ORs) among the treatments evaluated (empagliflozin, liraglutide, extended-release exenatide, dapagliflozin, dulaglutide, lixisenatide, canagliflozin, pioglitazone, DPP-4 inhibitors, subcutaneous semaglutide, sulphonylureas) [56]. Patients were being treated with metformin-based background therapy and were considered at high risk for a cardiovascular event [56]. The ORs for mortality in patients at high and low cardiovascular risk were 0.50 (95% Cl 0.31–0.83) and 0.58 (95% Cl 0.23–1.48), respectively [56].

### Cardiovascular clinical data with subcutaneous semaglutide

The subcutaneous (sc) presentation of semaglutide decreases the risk of MACEs in patients with diabetes. Subjects from the SUSTAIN 6 study treated with sc semaglutide had a 26% reduced risk of a cardiovascular events compared with the risk among individuals who received a placebo [57]. The protective effect was more notable for nonfatal stroke, with a 39% relative risk reduction. Nonfatal stroke occurred in 1.6% of patients treated with semaglutide and 2.7% treated with placebo (hazard ratio (HR) 0.61, 95% confidence interval (CI) 0.38–0.99, p = 0.04).

### Cardiovascular clinical data with pooled data from PIONEER 6 and SUSTAIN 6

Sc and oral formulations of semaglutide have been studied in a series of clinical trials in the SUSTAIN and PIONEER programs, respectively. However, there are no head-to-head studies comparing the two formulations. In a propensity score matching study, there was considerable overlap between the doses of oral semaglutide 7 and 14 mg and sc semaglutide 0.5 and 1.0 mg, respectively [58]. Population pharmacokinetic analysis indicated dose-proportional pharmacokinetic profiles for both oral and sc, with body weight being the main factor influencing exposure [58]. Similar exposure–response relationships were observed for efficacy (HbA1c and body weight) and tolerability (nausea and vomiting) of semaglutide, regardless of the route of administration [58]. A meta-analysis showed no statistically significant difference in efficacy between the two formulations at week 26, despite the numerically higher HbA1c response and body weight with sc semaglutide [59, 60].

A post hoc meta-analysis used a predictive model to evaluate the cardiovascular risk of patients from all PIONEER and SUSTAIN clinical trials (n = 18 studies) [61]. This analysis showed that semaglutide (both oral and sc) reduces the continuum relative and the absolute risk of MACE, especially in medium to high-risk patients, with no difference between trials evaluating only blood glucose control or cardiovascular events, and also with studies without active comparator, only versus placebo.

The post hoc analysis of patients only from the PIONEER 6 (evaluating oral semaglutide) and SUSTAIN 6 (evaluating sc semaglutide) studies found a 23.8% reduction in MACEs associated with semaglutide treatment (HR 0.76, 95% CI 0.62–0.92), especially in the prevention of nonfatal stroke (HR 0.65, 95% CI 0.43–0.97) [62].

Nonfatal stroke is a leading cause of disability worldwide, with high costs of medical care and a major impact on patients’ quality of life [63]. Stroke in patients with T2DM is associated with poor prognosis, high mortality, high incidence of neuromotor and neuropsychiatric sequelae, and high recurrence risk [64]. In addition to the lower incidence of nonfatal stroke in clinical trials [22, 40, 57], treatment with liraglutide or semaglutide (sc or oral) decreased the risk of dementia by 53% compared to the risk associated with placebo (HR 0.47, 95% CI 0.25–0.86) according to a pooled post hoc analysis of the LEADER, SUSTAIN 6 and PIONEER 6 trials, including 15,820 patients with T2DM [65].

Moreover, sc semaglutide showed neuroprotective activity in a stroke animal model, improved motor control and muscle strength, and reduced infarct volume, loss of neurons, and inflammation [66]. The mechanism is still unknown; experimental studies with other GLP-1 analogues (exendin-4 and liraglutide) have shown evidence of inhibition of oxidative stress, decreased apoptosis and prevalence of injured cells, decreased vascular proliferation, and increased _c_AMP levels in neurons and anti-inflammatory activity in microglial cells [67].

### Cardiovascular clinical data with oral semaglutide versus others GLP-1 receptor agonists

Seven different GLP-1 receptor agonists have CVOTs results (see Table 1). Three of them already have a cardiovascular indication approved. Oral semaglutide shows no significant difference compared with other GLP-1 receptor agonists in the incidence of MACE, in hospitalization for heart failure, and no difference on cardiovascular death (except for Lixisenatide: HR 0.5; CI 95% 0.26–0.96) [68].

**Table 1:** GLP-1 receptor agonists: cardiovascular indications and CVOTs results

GLP-1 receptor agonists	Semaglutide		Lixisenatide	Exenatide	Liraglutide	Dulaglutide	Albiglutide^a^
Administration route	Oral	Subcutaneous	Subcutaneous	Subcutaneous	Subcutaneous	Subcutaneous	Subcutaneous
Cardiovascular indication	No	Yes Reduction of MACEs in adults with T2DM and established CVD	No	No	Yes Reduction of MACEs in adults with T2DM and established CVD	Yes Reduction of MACEs in adults with T2DM and established CVD or multiple CV risk factors	No
CVOT [reference]	PIONEER 6 [40]	SUSTAIN 6 [57]	ELIXIA [106]	EXSCEL [107]	LEADER [22]	REWIND [108]	HARMONY [54]
Study population	3183 T2DM patients with established CVD	3297 T2DM patients with established CVD	6068 T2DM patients with acute coronary event in the last 180 days	14,752 T2DM patients with and without established CVD	9340 T2DM patients with established CVD	3183 T2DM patients with established CVD	9463 T2DM patients with established CVD
Intervention	Oral semaglutide 14 mg once a day vs. placebo	Semaglutide 0.5–1.0 mg sc once a week vs. placebo	Lixisenatide 20 μg sc once a day vs. placebo	Exenatide 2.0 mg sc once a week vs. placebo	Liraglutide 1.8 mg sc once a day vs. placebo	Dulaglutide 1.5 mg sc once a week vs. placebo	Albiglutide 30–50 mg sc once a week vs. placebo
Median follow-up	15.9 months	2.1 years	25 months	3.2 years	3.8 years	5.4 years	1.6 years
Primary endpoint: HR; 95%CI; superiority p-value	0.79; 0.57–1.11; p = 0.17	0.74; 0.58–0.95; p = 0.02	1.02; 0.89–1.17; p = 0.81	0.91; 0.83–1.00; p = 0.06	0.87; 0.78–0.97; p = 0.01	0.88; 0.79–0.99; p = 0.026	0.78; 0.68–0.90; p = 0.0006

*CV* cardiovascular, *CVD* cardiovascular disease, *CVOT* cardiovascular outcome trial, *GLP-1* glucagon-like peptide 1, *HR* hazard ratio, *MACE* major cardiovascular events, *SC* subcutaneous, *T2DM* type 2 diabetes mellitus, *Vs* versus

^a^Not currently available on the market

### Anti-atherogenic mechanisms of semaglutide

Studies to elucidate the cardiovascular protection mechanisms of GLP-1 have been conducted over the years, but they are not completely understood. Human recombinant GLP-1 and analogues have direct and indirect effects that are correlated with antiatherogenic properties, acting on signalling pathways in vascular smooth muscle cells [69]. GLP-1 reduces intracellular ROS, prevents oxidative stress injury, and increases cellular protection in arterial endothelial and smooth muscle cells [70, 71]. It promotes arterial vasodilation by binding GLP-1R in endothelial cells and releasing NO [69]. GLP-1 reduces endoplasmic reticulum stress and apoptosis induced by hyperglycaemia and regulates mitochondrial function via stimulation of optic atrophy protein 1 [69]. It improves endothelial function and promotes arterial vasodilatation in T2DM patients with ASCVD [29, 72].

GLP-1 inhibits macrophage foam cell formation, preventing the development of atherosclerotic plaques [73]. SC semaglutide significantly attenuates aortic plaque lesions in nondiabetic low-density-lipoprotein-receptor-deficient mice in a dose-independent manner and affects genes related to atherosclerosis [50]. It also reduces proatherogenic inflammation, decreasing plasma levels of the inflammatory cytokines TNF-α and IFN-γ and immune cell recruitment [50]. Data from the PIONEER programme show a clinically meaningful reduction in systemic inflammation with oral semaglutide, measured by C-reactive protein [35, 36]. Visceral fat accumulation is also frequent in T2DM patients and increases atherosclerosis and cardiometabolic risk [74]. SC semaglutide reduces the epicardial adipose tissue of patients with T2DM and obesity by 20% after 12 weeks of treatment [75].

Lipids play a significant role in atherosclerotic plaque formation. GLP-1 inhibits the postprandial increase in triglycerides (TGs) and free fatty acids in patients with diabetes [76]. Treatment with oral semaglutide improves the fasting lipid level profile, as exploratory analysis resulted in a statistically superior reduction in total cholesterol [35, 38, 42], low-density lipoprotein (LDL) [35, 42], triacylglycerols (TAGs) [35, 38, 39], and very-low-density lipoprotein (VLDL) compared to the effects of placebo [38]. Compared with active drugs, oral semaglutide was superior to empagliflozin in reducing total cholesterol and LDL [36] and was superior to sitagliptin in reducing total cholesterol, LDL, and TAGs [37, 41], but there was no significant difference when compared with another GLP-1 analogue (liraglutide) [38]. Studies in mice and humans showed that GLP-1 liraglutide acts directly and indirectly in LDL and VLDL catabolism, increases lipoprotein lipase gene expression responsible for TAG hydrolysis, and reduces apolipoprotein B48, diacylglycerol *O*-acyltransferase 1, and microsomal transfer protein gene expression, all of which are involved in chylomicron synthesis [77, 78]. Liraglutide reduces proprotein convertase subtilisin/kexin type 9, which interferes with LDL clearance, and retinol-binding protein 4, which is related to insulin resistance [77, 78]. It also suppresses oxidized LDL action by restoring the expression of Kruppel-like transcription factor 2, an important regulator of endothelial function, by improving endothelial hyperpermeability and by reducing vascular adhesion molecule expression [79].

Moreover, oral semaglutide improves systolic blood pressure, with superior reduction compared with placebo and sitagliptin [35, 37, 40, 42], adding an extra contribution to reduce cardiovascular risk.

### Blood glucose levels and atherosclerosis

One of the main strategies to reduce ASCVD risk in T2DM patients is to achieve blood glucose level control [44, 45]. Oral semaglutide showed superiority over placebo [35], empagliflozin [36], and sitagliptin [37] and noninferiority over liraglutide [38] in reducing HbA1c levels in T2DM patients. However, as noted previously by Zweck et al. [80], the normalization of HbA1c induced by oral semaglutide is directly associated with the reduction in cardiovascular risk, but it may not be related simply to improvement in glycaemic control; it may reflect drug class–mediated activation of other cardiovascular protective mechanisms. In fact, their analyses indicate that the cardiovascular efficacy of albiglutide is not driven by glycated haemoglobin [80]. An ongoing phase 3 trial, the SELECT study, will evaluate the cardiovascular benefit of semaglutide beyond blood glucose control, as the study population is composed of individuals who are overweight or obese without diabetes [81].

Oral semaglutide is associated with a low risk of hypoglycaemia because it stimulates insulin secretion according to glucose plasma levels, making this drug suitable for elderly individuals [82]. In the PIONEER programme [35–42], the most frequent adverse events were nausea and diarrhoea, which are common side effects of GLP-1 analogues. There were few hypoglycaemic events and were generally associated with sulfonylureas and insulin background therapy [35–42].

### Body weight and atherosclerosis

Obesity is a significant risk factor for T2DM and an independent risk factor for ASCVD [45]. Approximately 90% of T2DM patients are overweight or obese [83]. Obesity increases inflammation and endothelial dysfunction and is associated with hypertension, dyslipidaemia, and glucose intolerance [84, 85]. Subclinical coronary atherosclerosis is strongly associated with obesity [86].

Body weight reduction decreases cardiovascular risks and can result in remission of T2DM [87]. Significant weight loss (at least 10% of body weight) reduces HbA1c, blood pressure, lipid levels [88], and the incidence of MACEs including cardiovascular death, nonfatal myocardial infarction, nonfatal stroke, and hospitalized angina by up to 21% in T2DM patients; moreover, significant weight loss reduces the requirement for procedures, such as coronary artery bypass grafting, carotid endarterectomy, and percutaneous coronary intervention, as well as peripheral vascular disease, and total mortality by up to 24% [89, 90]. Even minimal weight loss (1 kg) decreases the risk of heart failure in T2DM patients by 5.9% [90].

Patients treated with oral semaglutide experienced clinically significant weight loss, as well as a reduction in body mass index and waist circumference [35–40]. Waist circumference is a reliable indicator of abdominal obesity, and it is considered a risk factor for ASCVD independent of body mass index [91]. Treatment with oral semaglutide reduced mean body weight within the first 14 weeks of treatment, and weight loss was generally maintained throughout the trials. Oral semaglutide was superior to placebo, sitagliptin, and liraglutide and similar to empagliflozin, with losses up to 4.44 kg compared with 0.5–3.6 kg for active-comparator arms and 0.4–1.4 kg compared with placebo [35–40]. When considering study drug discontinuation or use of rescue medication (trial product estimand results), the weight loss resulting from the use of oral semaglutide was up to − 5 kg, while that associated with other active comparators ranged from − 0.8 to − 3.8 kg, and that associated with placebo ranged from − 0.1 to + 0.6 kg [35–40]. Oral semaglutide significantly decreases craving for food more than empagliflozin [36, 37], and weight loss improves patients’ quality of life [37, 42]. The mechanisms underlying these results are based on the activity of GLP-1 in controlling eating, acting on satiety signals [92] in areas of the brain involved in food intake regulation [93] and peripheral action, reducing gastric emptying and intestinal motility, and slowing absorption [94].

### Therapeutic strategy and future perspectives of oral semaglutide

Given the prevalence of ASCVD in patients with diabetes [8], it is clear that GLP-1 analogues are underused. We currently have dulaglutide, liraglutide and sc semaglutide with approved CV indication (see Table 1). However, they are all in injectable form. Oral semaglutide benefits patients with high cardiovascular risk and is well accepted by patients [41]. Subjects from the PIONNEER 7 study showed comparable satisfaction between oral semaglutide and sitagliptin [41], a dipeptidyl peptidase-4 inhibitor (DPP-4i) largely used in monotherapy or combination when first-line treatment does not achieve adequate glycaemic control, but sitagliptin does not provide any cardiovascular benefit [95].

Oral presentation improves patients’ adherence, especially in chronic diseases. According to results from a multinational survey interviewing 3742 diabetic patients, an oral antidiabetic drug is preferable as the first choice of medication and medications for long-term use, especially for those with high HbA1c and comorbidities (obesity, hypertension, dyslipidaemia) [96]. Treatment satisfaction is directly related to adherence [97]. Patients from the PIONEER trials had higher satisfaction with oral semaglutide [38, 41, 42] than with placebo [38, 39, 42] and sitagliptin [41], especially in regard to hyperglycaemia. Moreover, preliminary data from real-world studies show a better metabolic control of T2DM patients with oral semaglutide, which is related to good treatment adherence [98, 99].

The best timing for introducing oral semaglutide has not yet been established. It is important to be more efficient in T2DM treatment starting from the time of diagnosis. In the Verify study, patients received a DPP4i, vildagliptin, along with metformin as an early treatment [100]. This early combination therapy with drugs targeting different actions provided better blood glucose control, decreased treatment failure, and extended the time to initiating insulin therapy [100]. It is expected that early combination therapy modifies the natural history of the disease. We could expect from early treatment with oral semaglutide and other GLP-1 analogues a better control of cardiovascular risks as a result of its actions exerted before atherosclerosis develops, in addition to the benefits of superior glucose control and weight loss.

Non-alcoholic steatohepatitis (NASH) is highly prevalent in T2DM patients, with high morbidity and mortality [101]. Compared to individuals without steatosis, patients with NASH have an increased incidence of ASCVD (HR 1.37, 95% CI 1.10–1.72) [102] and fatal and nonfatal MACEs (OR: 1.64, 95% CI 1.26–2.13) [103]. The physiopathology of NASH involves genes that increase plasma lipids and release procoagulant and proinflammatory factors, resulting in a higher risk for cardiovascular events [104]. Semaglutide benefits patients with NASH. In a preclinical study, sc semaglutide reduced hepatic TAGs and decreased the expression of 3 of 5 collagen genes and other inflammatory markers responsible for the development of liver fibrosis [50]. A phase 2 clinical study showed resolution of NASH and no worsening of fibrosis in 59% of patients treated with a higher dose of sc semaglutide compared with 17% of patients treated with placebo (p < 0.001) [105]. The oral presentation has not yet been studied for this indication, but encouraging evidence should be expected in the future. The impact of NASH treatment on ASCVD is still unknown.

## Conclusion

The oral GLP-1 analogue semaglutide is a new drug that adds several benefits to diabetes treatment in addition to blood glucose control. It has the advantages of oral use, weight reduction, and potential positive cardiovascular effects in clinical practice. The antiatherogenic effect of the GLP-1 class is widely described in the literature, but we still need more clinical evidence on the cardiovascular impact in patients with diabetes. Data on the long-term efficacy of oral semaglutide for the significant reduction in MACEs are expected, as clinical studies are already being conducted. This medication is a safe option and should be part of clinicians’ arsenal to decrease cardiovascular risk in T2DM patients.

## Supplementary Information

**Additional file 1:** **Table S1. **Cardiovascular events reported during the PIONEER program with oral semaglutide.

## Data Availability

Not applicable.

## Abbreviations

ASCVD: Atherosclerotic cardiovascular disease
CHD: Coronary heart disease
CI: Confidence interval
CVD: Cardiovascular disease
CVOT: Cardiovascular outcome trial
DPP-4i: Dipeptidyl peptidase-4 inhibitor
GLP-1: Glucagon-like peptide 1
GLP-1R: Glucagon-like peptide 1 receptor
HbA1c: Glycated haemoglobin
HR: Hazard ratio
LDL: Low-density lipoprotein
MACEs: Major cardiovascular events
NO: Nitric oxide
OR: Odds ratio
PIONEER: Peptide Innovation for Early Diabetes Treatment
ROS: Reactive oxygen species
SC: Subcutaneous
SNAC: Sodium *N*-[8-(2-hydroxybenzoyl)amino] caprylate
TAGs: Triacylglycerols
TGs: Triglycerides
T2DM: Type 2 diabetes mellitus
VLDL: Very-low-density lipoprotein
