# Supplementary material for: Oral GLP-1 analogue: perspectives and impact on atherosclerosis in type 2 diabetic patients
Source: Cardiovasc Diabetol. 2021 Dec 15;20:235. doi: 10.1186/s12933-021-01417-0 (PMC8675489; doi:10.1186/s12933-021-01417-0)
Supplement: Supplementary file 1 — Additional file 1: Table S1. Cardiovascular events reported during the PIONEER program with oral semaglutide. [file 12933_2021_1417_MOESM1_ESM.docx]

**Table 1. Cardiovascular events reported during the PIONEER program with oral semaglutide.**

| Study  [Reference] | Design | Population* | Treatment arms | Change in HbA1c | EDT  (95% CI) | Safety**: cardiovascular events  Percentage of patients (number of events) | | | | |
| --- | --- | --- | --- | --- | --- | --- | --- | --- | --- | --- |
|  | | | | | | **CV events** | **Acute coronary syndrome** | **Cerebrovascular events** | **Heart failure requiring hospitalization** | **CV death** |
| PIONEER 1 [35] | Randomized  double-blind,  placebo controlled,  parallel, 26 weeks | n= 703  T2DM uncontrolled with diet and exercise | Semaglutide  3mg | -0.8% | -0.7 % (-0.9 to -0.5) p<0.001 | 0 | 0 | 0 | 0 | 0 |
|  |  |  | Semaglutide  7mg | -1.3% | -1.2% (-1.5 to -1.0)  p<0.001 | 0 | 0 | 0 | 0 | 0 |
|  |  |  | Semaglutide  14mg | -1.5% | -1.4% (-1.7 to -1.2)  p<0.001 | 0.6 (1) | 0.6 (1) | 0 | 0 | 0.6 (1) |
|  |  |  | Placebo | -0.1% | - | 1.1 (2) | 0 | 1.1 (2) | 0 | 0 |
| PIONEER 2 [36] | Randomized, open label, 52 weeks | n= 822  T2DM uncontrolled on  metformin | Semaglutide 14mg | -1.4% | -0.5% (-0.7 to -0.4)  <0.0001 | 1.2 (5) | 1.0 (4) | 0 | 0.5 (2) | 0 |
|  |  |  | Empaglifozin 25mg | -0.9% |  | 1.5 (6) | 0.2 (1) | 1.0 (4) | 0.2 (1) | 0 |
| PIONEER 3 [37] | Randomized,  double-blind,  double dummy  parallel group, 78 weeks | n= 1864  T2DM uncontrolled with metformin and/or sulfonylurea | Semaglutide  3mg | -0.5% | 0.2% (0.1 to 0.4)  <0.001 | 3.2 (15) | 0.9 (4) | 1.5 (7) | 0.9 (4) | 0.4 (2) |
|  |  |  | Semaglutide  7mg | -1.1% | -0.3% (-0.4 to -0.2)  <0.001 | 1.5 (7) | 0.9 (4) | 0.2 (1) | 0 | 0 |
|  |  |  | Semaglutide  14mg | -1.4% | -0.6% (-0.7 to -0.5)  <0.001 | 1.1 (5) | 0.2 (1) | 0.4 (2) | 0.2 (1) | 0.2 (1) |
|  |  |  | Sitagliptin 100mg | -0.8% | - | 2.1 (10) | 0.9 (4) | 0.4 (2) | 0.6 (3) | 0 |
| PIONEER 4 [38] | Randomized,  double-blind,  double dummy, 52 weeks | n= 711  T2DM  on metformin  with or without an SGLT2 inhibitor | Semaglutide 14mg | -1.3% | -0.2% (-0.3 to -0.1)  P=0.0056*** | 1.4 (5) | 0.4 (1) | 0.7 (2) | 0 | 0.7 (2) |
|  |  |  |  |  | -1.2% (-1.4 to -1.0)  P<0.001**** |  |  |  |  |  |
|  |  |  | Liraglutide 1.8mg | -1.1% | - | 1.1 (4) | 0.4 (1) | 0 | 0.4 (1) | 0.7 (2) |
|  |  |  | Placebo | -0.1% | - | 1.4 (2) | 0.7 (1) | 0.7 (1) | 0 | 0 |
| PIONEER 5 [39] | Randomized, double blind,  26 weeks | n= 324  T2DM and moderate renal impairment on metformin and/or sulfonylurea, or basal insulin | Semaglutide 14mg | -1.1% | 1.0% (-1.2 to -0.8)  p<0.0001 | 3.1 (5) | 1.8 (3) | 1.2 (2) | 0 | - |
|  |  |  | Placebo | -0.1% |  | 1.2 (2) | 0 | 0 | 0.6 (1) | - |
| PIONEER 6 [40] | CVOT Randomized,  double-blind,  placebo controlled,  80 weeks | n=3,183  T2DM and established CVD | Semaglutide 14mg | -1.0% | - | 6.1(97) | 3.0 (48) | 0.8 (12) | 1.3 (21) | 0.9 (15) |
|  |  |  | Placebo | -0.3% | - | 7.0 (111) | 2.3 (38) | 1.0 (16) | 1.5 (24) | 1.9 (30) |
| PIONEER 7 [41] | Randomized, open label, 52 weeks | n=504  T2DM inadequately controlled on of one or two oral glucose-lowering drugs | Semaglutide with flexible dose adjustment | 63% | - | 0.4 (1) | 0.4 (1) | 0 | 0 | 0 |
|  |  |  | Sitagliptin 100mg | 28% | p<0.0001 | 1.6 (4) | 0.4 (1) | 0.4 (1) | 0.4 (1) | 0.8 (2) |
| PIONEER 8 [42] | Randomized, open label, 26 weeks | n= 731 T2DM under insulin therapy with or without  metformin | Semaglutide 3mg | -0.6% | -0.6 (-0.7 to -0.4)  p<0.0001 | 2.2 (4) | 1.1 (2) | 1.1 (2) | 0.5 (1) | 0 |
|  |  |  | Semaglutide 7mg | -1.0% | -1.0 (-1.2 to -0.8) p<0.0001 | 2.8 (5) | 0.6 (1) | 1.7 (3) | 1.1 (2) | 0 |
|  |  |  | Semaglutide 14mg | -1.4% | -1.4 (-1.6 to -1.2)  p<0.0001 | 2.8 (5) | 1.2 (2) | 0.6 (1) | 0 | 0 |
|  |  |  | Placebo | -0.0% | - | 2.7 (5) | 1.0 (2) | 1.6 (3) | 0 | 0 |

*Randomized

**data confirmed by an external adjudication committee

*** versus liraglutide

**** versus placebo

CV: cardiovascular. CI: confidence interval. EDT: estimated treatment differences. T2DM: type 2 diabetes mellitus.
